# Dynamics of NT-proBNP in Pregnancy: Why Values May Be Elevated in the First Trimester∗

**DOI:** 10.1016/j.jacadv.2023.100287

**Published:** 2023-03-31

**Authors:** Amy A. Sarma, Nandita S. Scott

**Affiliations:** Division of Cardiology, Massachusetts General Hospital, Boston, Massachusetts, USA

**Keywords:** NT-proBNP, preeclampsia, pregnancy

**Figure:**
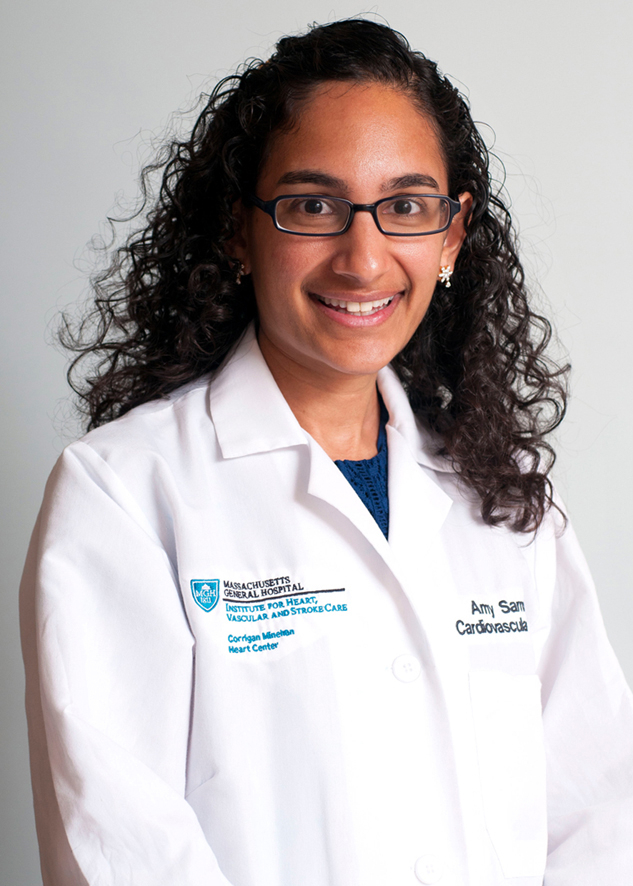

Pregnancy is a time of rapid hemodynamic and hormonal change. Starting in early pregnancy, maturation of the low resistance placenta results in a decrease in systemic vascular resistance and mean arterial pressure.^1^ Cardiac output significantly increases to accommodate increasing plasma volume. Simultaneous with these changes, individuals often experience symptoms even in normal pregnancy that are challenging to distinguish from those of early cardiovascular decompensation, including dyspnea, fatigue, and lower extremity edema. Individuals are also at significantly higher risk for adverse cardiovascular events during pregnancy as compared to the non-pregnant state. As such, there has been significant interest in tools to assist in the identification of pregnant individuals who may merit further cardiovascular testing when presenting with suspected symptoms. As cardiac biomarkers are commonly used for this purpose in the non-pregnant population, there is growing interest in understanding their potential utilization in pregnancy.

N-terminal pro-B-type natriuretic peptide (NT-proBNP) is released in response to cardiomyocyte stretch and now widely utilized in the assessment of heart failure among non-pregnant patients. Despite the marked hemodynamic changes of pregnancy, studies to date of relatively modest size have demonstrated that while NT-proBNP levels are higher during pregnancy than non-pregnant values, they generally remain low throughout the trimesters in normal pregnancy.^2^ As such, NT-proBNP retains its negative predictive value. Because elevations often occur in the context of complications like hypertensive disorders of pregnancy (HDP), peripartum cardiomyopathy, and heart failure due to structural disease or diastolic dysfunction, those presenting with symptoms of suspected heart failure and elevated NT-proBNP merit further cardiovascular evaluation.^2^ However, in the context of abrupt hemodynamic changes that occur with delivery, natriuretic peptides can transiently increase among pregnant individuals early postpartum even in the absence of overt cardiovascular dysfunction.^2^, ^3^, ^4^ Therefore, based on prior data, NT-proBNP levels are not always associated with clinical heart failure, particularly in the early postpartum period.

More recently, observational studies have also suggested that NT-proBNP levels may be elevated in early pregnancy and that these elevations may actually be normative. A recent study of 260 pregnant individuals reported a 95% upper reference limit for NT-proBNP in the first and second trimesters of 200 pg/mL, as compared with an upper limit of 150 pg/mL in the third trimester.^5^ In this issue of *JACC: Advances*, Minhas et al^6^ add to this literature by reporting on NT-proBNP in a large, nationally representative sample from the National Health and Nutrition Examination Survey of 2,134 patients (546 pregnant) aged 20 to 40 years without a self-reported history of cardiovascular disease. Authors found a 20% incidence of elevated NT-proBNP (≥125 pg/mL) among first trimester samples, as compared with 2% in the third trimester and 8% among non-pregnant patients. The finding of higher first trimester levels persisted after adjustment for demographics and cardiovascular risk factors.

These data add to a growing body of observational studies that NT-proBNP is higher in normal early pregnancy as compared with third trimester. Data from a recent cohort study of 4,103 pregnant patients take this observation further in reporting that higher NT-proBNP concentrations in early pregnancy were actually associated with a lower risk of developing HDP, as well as hypertension 2 to 7 years postpartum.^7^ Similarly, Minhas et al found an inverse relationship between systolic blood pressure and NT-proBNP.

Why NT-proBNP seems to be higher in early pregnancy as compared with third trimester still lacks a definitive mechanistic explanation, but many hypotheses have been proposed by Minhas et al and others. It may be due to early hormonal changes as estrogen stimulates the cardiac natriuretic system. Alternatively, greater renal filtration in later pregnancy may account for lower NT-proBNP. However, the observation that lower NT-proBNP levels may actually correlate with increased risk of HDP from Hauspurg et al,^7^ raise more interesting hypotheses that higher NT-proBNP levels may reflect normative and appropriate early-pregnancy volume expansion to enable adequate uteroplacental flow, and reduced vascular resistance. In contrast, patients who develop HDP experience abnormal placentation in early pregnancy, impaired volume expansion, and vascular stiffness which may be reflected in lower NT-proBNP levels in the first trimester.

The hypotheses raised by this latest manuscript and others merit further investigation in studies that couple biomarker values with cardiovascular structural and functional data, as well as clinical outcomes. In particular, understanding if and how NT-proBNP or other biomarkers may enable the identification of at-risk individuals exhibiting early vascular stiffness, adverse cardiac remodeling, and abnormal hemodynamic adaptation would be highly valuable in identifying patients who merit closer monitoring and assessment for HDP and cardiovascular events. In current clinical practice, however, NT-proBNP remains most useful for its negative predictive value in reducing the probability of a diagnosis heart failure among pregnant patients presenting with suspected symptoms (Figure 1). However, the implications of NT-proBNP elevations in the absence of evident cardiovascular dysfunction remains poorly understood, may differ based on timing (early pregnancy vs early postpartum), and is an important question for future research.

**Figure 1:**
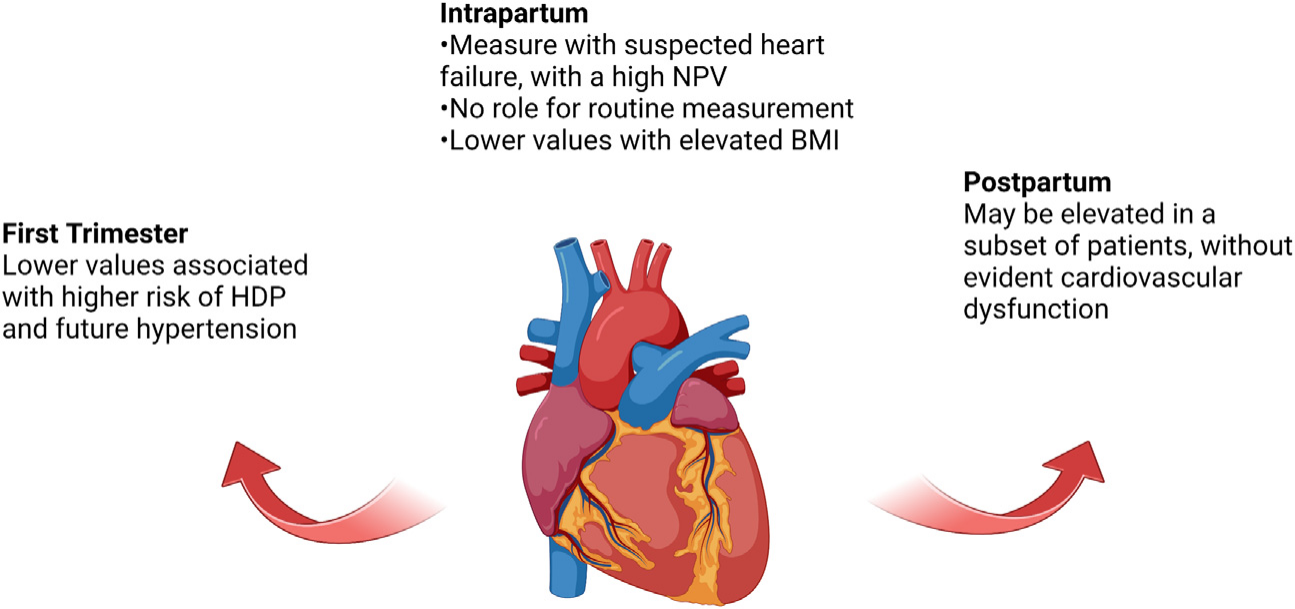
**Utilization of NT-proBNP in Pregnancy: Clinical Considerations** BMI = body mass index; HDP = hypertensive disorders of pregnancy; NPV: negative predictive value; NT-proBNP = N-terminal pro-B-type natriuretic peptide.

## Funding support and author disclosures

Dr Sarma has received funding from the CRICO Patient Safety grant; and has a consulting relationship with Pfizer (which is not related to the current work). Dr Scott has reported that she has no relationships relevant to the contents of this paper to disclose.
